# Multilevel Intersectional Social Drivers of Health and Nulliparous Term, Singleton, Vertex Cesarean Delivery

**DOI:** 10.1097/og9.0000000000000192

**Published:** 2026-07-30

**Authors:** Jameaka L. Hamilton, William A. Grobman, Jiqiang Wu, Lynn M. Yee, David M. Haas, Becky Mcneil, Jessica Wilder, Hyagriv Simhan, Uma M. Reddy, Robert M. Silver, Lisa Levine, George Saade, Jun Wu, Courtney D. Lynch, Kartik K. Venkatesh

**Affiliations:** Department of Obstetrics and Gynecology, The Ohio State University, Columbus, Ohio; Department of Obstetrics and Gynecology, Brown University, Providence, Rhode Island; Department of Obstetrics and Gynecology, Northwestern University, Chicago, Illinois; Department of Obstetrics and Gynecology, Indiana University, Indianapolis, Indiana; RTI International, Durham, North Carolina; Department of Obstetrics and Gynecology, Case Western Reserve University, Cleveland, Ohio; Department of Obstetrics and Gynecology, University of Pittsburgh, Pittsburgh, Pennsylvania; Department of Obstetrics and Gynecology, Yale University, New Haven, Connecticut; Department of Obstetrics and Gynecology, University of Utah, Salt Lake City, Utah; Department of Obstetrics and Gynecology, University of Pennsylvania, Philadelphia, Pennsylvania; Department of Obstetrics and Gynecology, EVMS, Norfolk, Virginia; and Department of Environmental and Occupational Health, University of California, Irvine, Orange, California.

## Abstract

**OBJECTIVE:**

To examine the intersectionality of three frequently measured neighborhood social determinants during pregnancy—socioeconomic disadvantage, food access, and walkability—and their collective association with nulliparous term, singleton, vertex (NTSV) cesarean delivery.

**METHODS:**

This was a secondary analysis of data from the Nulliparous Pregnancy Outcomes Study: Monitoring Mothers-To-Be observational cohort. Home addresses in the first trimester were geocoded at the census-tract level to define three adverse neighborhood measures: 1) socioeconomic disadvantage by the Area Deprivation Index (in tertiles), 2) low food access by the U.S. Department of Agriculture *Food Access Research Atlas* (yes/no), and 3) low walkability by the Environmental Protection Agency National Walkability Score (yes/no). The exposure was the number of neighborhood adverse social determinants and was assessed by number (0, 1, 2 or more) and specific combinations. The outcome was NTSV cesarean delivery. Multivariable modified Poisson regression with robust error variance was used. We secondarily assessed the distribution of clinical indications for cesarean delivery and whether associations between neighborhood social determinants and cesarean delivery varied by self-reported race and ethnicity.

**RESULTS:**

Among 8,010 nulliparous individuals with NTSV deliveries, 27.3% lived in a neighborhood in the top tertile of socioeconomic disadvantage, 24.5% with low food access, and 66.4% with low walkability. The risk of NTSV cesarean delivery was higher only among individuals living in neighborhoods with low walkability (adjusted relative risk [RR] 1.17, 95% CI, 1.06–1.28). Individuals exposed to one (adjusted RR 1.14, 95% CI, 1.01–1.27) or two or more (adjusted RR 1.19, 95% CI, 1.06–1.34) compared with no adverse neighborhood social determinants had a higher risk of cesarean delivery. Individuals living in neighborhoods with low walkability and high socioeconomic disadvantage (adjusted RR 1.21, 95% CI, 1.03–1.41) or low walkability and low food access (adjusted RR 1.20, 95% CI, 1.05–1.37) had a higher risk of cesarean delivery. The frequency of all three adverse neighborhood social determinants was higher for non-Hispanic Black and Hispanic individuals compared with non-Hispanic White individuals (*P*<.05), but the above association between neighborhood social determinants and cesarean delivery did not vary by self-reported race and ethnicity (adjusted interaction *P*=.6).

**CONCLUSION:**

In a prospective U.S. cohort, living in a neighborhood with low walkability, alone and in combination with socioeconomic disadvantage and food access, was associated with an increased risk of NTSV cesarean delivery.

## Abstract 2

Living in neighborhoods with one or more adverse neighborhood social determinants compared with none in early pregnancy was associated with a higher risk of nulliparous term, singleton, vertex cesarean delivery.

Nearly 1 in 3 pregnant individuals had a cesarean delivery in the United States from 2009 to 2019, and since 2020, the overall cesarean delivery rate has increased.^1^ The main reason for the rise in cesarean delivery is an increase in nulliparous term, singleton, vertex (NTSV) cesarean delivery, which accounts for up to 60% of all cesarean deliveries.^1^ Over 80% of individuals with a primary cesarean delivery will later have a repeat cesarean delivery.^2^ The frequency of NTSV cesarean delivery for all indications is higher among racially and ethnically minoritized groups compared with non-Hispanic White individuals in the United States.^3^ Although cesarean delivery can improve perinatal outcomes when clinically indicated, it may also increase maternal morbidity with no improvement in perinatal outcomes when overused.^4^

Individuals who experience adverse social determinants of health (SDoH) are more likely to have a cesarean delivery, further contributing to disparate experiences of maternal morbidity and mortality.^5^ Adverse SDoH can be assessed at individual and neighborhood levels and potentially as a broader multidimensional constructs when combined together.^6^ Increasing data emphasize that neighborhood SDoH are associated with adverse pregnancy outcomes and that, in many cases, neighborhood SDoH may be stronger risk factors for adverse pregnancy outcomes than individual SDoH.^7^ Recent data highlight the association of three neighborhood SDoH measures measured individually with adverse pregnancy outcomes.^8–11^ These three measures include composite socioeconomic disadvantage including factors related to income, education, employment, and housing quality and two more specific constructs: walkability, which assesses how well community design supports pedestrians based on five main factors (dense population, diverse land use, connected street networks, destination accessibility, and transit proximity), and food access, which assesses food access indicators for low-income and other census tracts using different measures of supermarket accessibility. Neighborhood SDoH includes environmental factors that have been called the exposome such as physical, chemical, and social influences that influence human biology.

Each neighborhood factor in pregnancy does not occur in isolation, and it is possible that there may be particular combinations of these neighborhood SDoH factors that are associated with worse pregnancy outcomes.^12^ The collective association of multiple co-occurring neighborhood SDoH with pregnancy outcomes remains to be examined. We chose NTSV cesarean birth as the pregnancy outcome because it is an important patient-reported outcome that affects birth experience and is a health care quality metric. In addition, cesarean delivery reflects labor management practices, hospital-level practice patterns, variation in clinical judgment, access to care, and patient–clinician decision making that are affected by SDoH.

We investigated how the intersectional exposure of three neighborhood SDoH measures in early pregnancy—socioeconomic disadvantage, food access, and walkability—was associated with NTSV cesarean delivery. We secondarily assessed 1) the distribution of clinical indications for NTSV cesarean delivery among various combinations of neighborhood SDoH measures and 2) whether the association between neighborhood SDoH and cesarean delivery varied by self-reported race and ethnicity (effect modification) to examine how persistent obstetric health disparities related to the social construct of race and ethnicity may be related to neighborhood SDoH.

## METHODS

This analysis used data from the prospective nuMoM2b (Nulliparous Pregnancy Outcomes Study: Monitoring Mothers-to-Be) cohort. Individuals in this cohort were enrolled at eight U.S. medical centers from October 2010 to September 2013 (ClinicalTrials.gov NCT01322529). Full details of this study have been described previously.^13^ Data were collected prospectively by standardized patient interviews, surveys, and health record abstraction at up to three time points during pregnancy and at delivery. Approvals from the IRBs were obtained at each nuMoM2b study site. All participants provided written informed consent before participation.

Briefly, individuals met inclusion criteria for enrollment in the original study if they had no prior delivery at 20 weeks of gestation or later, had a viable singleton pregnancy in the first trimester at enrollment, and did not have a known lethal fetal malformation or fetal aneuploidy. The present analysis was restricted to individuals with NTSV pregnancies at delivery for whom exposure (three neighborhood SDoH measures) and outcome (delivery) data were available. We excluded participants if mode of delivery was missing or if participants did not provide a residential address (such as post office box number, rural address without geocode coordinates) that could be geocoded to define neighborhood SDoH measures.

We first defined three neighborhood SDoH measures: 1) socioeconomic disadvantage by the ADI (Area Deprivation Index), 2) food access by the U.S. Department of Agriculture *Food Access Research Atlas*, and 3) walkability by the Environmental Protection Agency National Walkability Score. Briefly, the ADI incorporates 17 U.S. Census indicators across income, education, employment, and housing quality domains to generate a composite score, which was then converted to a percentile to rank disadvantaged communities nationally. We analyzed ADI by national tertiles from low (tertile 1) to high (tertile 3) deprivation. Low food access (yes/no) provided a spatial overview of neighborhood-level indicators of food access defined by low income and low supermarket accessibility.^14,15^ Low walkability (yes/no) was defined based on a National Walkability Index using intersection density, proximity to transit stops, and employment and household mix, at a score of less than 15.26 per current guidance.^16^ To calculate each measure, the participant's primary residential address in the nuMoM2b study was collected through a structured interview at the first study visit from 6 to 13 weeks of gestation. Subsequently, addresses were geocoded with ArcGIS (www.arcgis.com) and linked at the Census tract level.

For this analysis, these three factors were modeled to reflect which neighborhood SDoH co-occurred for a given residential address. First, we analyzed the exposure as the number of adverse neighborhood SDoH experienced, expressed as no (reference), one, or two or more measures. The last category was created to allow meaningful analysis given the low frequency of experiencing all three neighborhood SDoH measures concurrently. We then assessed the specific types of combinations that existed (ie, exposure to one: only ADI, only walkability, only food access; or exposure to two: ADI and walkability, ADI and food access, or walkability and food access).

The primary outcome was NTSV cesarean delivery. We secondarily assessed five mutually exclusive primary clinical indications for NSTV cesarean delivery per current guidance: maternal (including maternal request, active herpes, or any hypertensive disorder), fetal (including nonreassuring fetal status, umbilical cord prolapse, or suspected macrosomia), obstetric (including arrest of dilation or descent, failed induction of labor, or unsuccessful operative vaginal delivery), placental or uterine (including placental abruption, prior uterine surgery [eg, myomectomy], placenta previa, or uterine anomaly), or other/no listed indication.^17^

We compared baseline sociodemographic and clinical characteristics by the three neighborhood SDoH measures using χ^2^ or Fisher exact test for categorical variables. Modified Poisson regression with robust error variance was used to model the association between combinations of neighborhood SDoH and cesarean delivery. We adjusted for maternal age at delivery (continuous), Medicaid insurance status (yes/no), body mass index (BMI; continuous), chronic hypertension (yes/no), and pregestational diabetes mellitus (yes/no). Confounding variables were selected for inclusion a priori according to a directed acyclic graph (Appendix 1, available online at https://links.lww.com/AOG/E775). Given the potential to adjust for individual SDoH factors that could be collinear or on the causal pathway when the exposure was a neighborhood SDoH factor, we used a causal framework that accounted for how exposure to neighborhood characteristics is related to individual-level health outcomes (ie, the contribution of exposure to neighborhood characteristics to variation between individuals in health).^18^

In secondary analyses, we assessed whether the association between the three neighborhood SDoH measures and cesarean delivery differed according to self-reported racial and ethnic identity to examine whether persistent obstetric health disparities related to the social construct of race and ethnicity may be related to neighborhood SDoH (effect modification). Self-reported race and ethnicity as a social construct was categorized as Hispanic, non-Hispanic Asian–Pacific Islander, non-Hispanic Black, non-Hispanic White, and multiracial or not identified by the participant. Race and ethnicity were categorized per the Centers for Disease Control and Prevention National Center for Health Statistics Natality Files to be consistent with public reporting of NTSV cesarean delivery rates. We added a term in the above multivariable model to assess for interaction between the primary exposure (neighborhood adverse social determinants) and the effect modifier (race and ethnicity) and, if the statistical interaction was significant (*P*<.05) a priori, then planned to conduct stratified analyses by race and ethnicity.

Multivariate imputation by chained equations was used for imputation of all covariates with missing data with 30 imputed sets in which the model was fit in each imputed set and then combined across all 30 imputed sets; data were assumed to be missing at random. All statistical analyses were performed with R version 4.2.0.

## RESULTS

Of the 10,038 individuals enrolled in the prospective nuMoM2b cohort, 915 (9.1%) were nonvertex deliveries and 770 (8.4%) delivered before 37 weeks and were excluded. Of the remaining 8,353 individuals with term vertex deliveries, 341 (4.1%) were missing geocoded neighborhood SDoH exposure and 2 were missing delivery outcome data (n=2, 0%), resulting in 8,010 individuals (95.9%) eligible for this secondary analysis. Individuals who were excluded were more likely to receive Medicaid insurance, self-report minoritized race and ethnic identity, have lower educational attainment, report household poverty, report tobacco use, and have a higher BMI and chronic hypertension (*P*<.05 for all, Appendix 2, https://links.lww.com/AOG/E775).

Overall, the median age was 27 years (interquartile range 22–31 years); 27.1% had Medicaid insurance; 15.0% lived below the federal poverty level; 12.9% self-identified as non-Hispanic Black; and 16.8% self-identified as Hispanic. The median BMI was 24.7 (interquartile range 22.0–29.2); 2.3% had chronic hypertension; and 1.1% had pregestational diabetes.

The median ADI was 44.8 (interquartile range 18–71); 27.3% lived in the top (or worst) ADI tertile; 24.4% lived in a neighborhood with low food access; and 66.2% lived in a neighborhood with low walkability. Regarding the intersection of the three neighborhood SDoH measures, 23.4% experienced no adverse neighborhood SDoH, 39.5% experienced only one adverse neighborhood SDoH measure, 33.0% experienced only two adverse neighborhood SDoH measures, and 4.1% experienced all three adverse neighborhood SDoH measures; 37.2% experienced two or more adverse neighborhood SDoH measures (Table 1). Sociodemographic or individual SDoH characteristics varied according the number of adverse neighborhood SDoH experienced (Table 1). For example, those who lived in areas with a greater number of neighborhood SDoH were more likely to receive Medicaid insurance and have lower educational attainment (*P*<.05).

**Table 1.** Sociodemographic and Clinical Characteristics of Study Population Overall and by Three Neighborhood-Level Social Determinants of Health Measures (N=8,010)*

Row Percentage	Overall	No Neighborhood SDoH (N=1,871)	Only 1 Neighborhood SDoH (N=3,162)	2 or More Neighborhood SDoH (N=2,977)
Neighborhood SDoH measures				
ADI				
Tertile 1 (least deprivation)	3,605 (45.0)	1,301 (36.1)	1,433 (39.8)	871 (24.2)
Tertile 2	2,216 (27.7)	570 (25.7)	1,098 (49.5)	548 (24.7)
Tertile 3 (most deprivation)	2,189 (27.3)	0 (0.0)	631 (28.8)	1,558 (71.2)†
Low walkability				
No	2,707 (33.8)	1,871 (69.1)	768 (28.4)	68 (2.5)
Yes	5,303 (66.2)	0 (0.0)	2,394 (45.1)	2,909 (54.9)†
Low food access				
No	6,054 (75.6)	1,871 (30.9)	3,025 (50.0)	1,158 (19.1)
Yes	1,956 (24.4)	0 (0.0)	137 (7.0)	1,819 (93.0)†
Participant characteristics				
Age (y)				
17 or younger	187 (2.3)	16 (8.6)	54 (28.9)	117 (62.6)
18–34	7,118 (88.9)	1,609 (22.6)	2,812 (39.5)	2,697 (37.9)
35–39	600 (7.5)	208 (34.7)	261 (43.5)	131 (21.8)
40 or older	105 (1.3)	38 (36.2)	35 (33.3)	32 (30.5)†
Medicaid insurance (N=7,959)				
No	5,801 (72.9)	1,643 (28.3)	2,341 (40.4)	1,134 (52.5)
Yes	2,158 (27.1)	223 (10.3)	801 (37.1)	1,817 (31.3)†
Self-identified race and ethnicity				
Non-Hispanic Asian	324 (4.0)	126 (38.9)	135 (41.7)	63 (19.4)
Non-Hispanic Black	1,030 (12.9)	90 (8.7)	357 (34.7)	583 (56.6)
Hispanic	1,343 (16.7)	229 (17.1)	618 (46.0)	496 (36.9)
Non-Hispanic White	4,916 (61.4)	1,329 (27.0)	1,917 (39.0)	1,670 (34.0)
Nonspecified identity	397 (5.0)	97 (24.4)	135 (34.0)	165 (41.6)†
Education (n=8,004)				
High school or less	588 (7.3)	49 (8.3)	175 (29.8)	364 (61.9)
Some college	2,441 (30.5)	294 (12.0)	999 (40.9)	1,148 (47.0)
College graduate	3,093 (38.6)	780 (25.2)	1,232 (39.8)	1,081 (34.9)
Graduate degree	1,882 (23.5)	748 (39.7)	753 (40.0)	381 (20.2)†
Tobacco uses (n=8,005)				
No	6,637 (82.9)	1,663 (25.1)	2,655 (40.0)	2,319 (34.9)†
Yes	1,368 (17.1)	206 (15.1)	506 (37.0)	656 (48.0)
BMI (n=7,865)				
Underweight	183 (2.3)	40 (21.9)	71 (38.8)	72 (39.3)
Normal weight	4,064 (51.7)	1,138 (28.0)	1,609 (39.6)	1,317 (32.4)
Overweight	1,940 (24.7)	411 (21.2)	772 (39.8)	757 (39.0)
Obesity	939 (11.9)	178 (19.0)	371 (39.5)	390 (41.5)
Severe obesity	739 (9.4)	88 (11.9)	273 (36.9)	378 (51.2)†
Household income and size relative to the U.S. poverty level (n=6,590) (%)				
Less than 130	4,655 (70.6)	1,494 (32.1)	1,859 (39.9)	1,302 (28.0)
130–350	948 (14.4)	133 (14.0)	379 (40.0)	436 (46.0)
More than 350	987 (15.0)	96 (9.7)	348 (35.3)	543 (55.0)†
Pregestational diabetes				
No	7,922 (98.9)	1,860 (23.5)	3,129 (39.5)	2,933 (37.0)
Yes	88 (1.1)	11 (12.5)	33 (37.5)	44 (50.0)†
Chronic hypertension (n=8,001)				
No	7,840 (98.0)	1,849 (23.6)	3,091 (39.4)	2,900 (37.0)†
Yes	161 (2.0)	22 (13.7)	67 (41.6)	72 (44.7)

SDoH, social determinants of health; ADI, Area Deprivation Index; BMI, body mass index.

Data are n (%) unless otherwise specified.

* The χ^2^ test was used to compare categorical variables, and Wilcoxon rank-sum test was used for continuous variables.

† *P*<.05 for assessed characteristics above.

The overall frequency of NTSV cesarean delivery was 24.2%. The most frequent indication for cesarean delivery was an obstetric cause (n=1,118, 57.5%), which included arrest of dilation (n=618, 31.8%), arrest of descent (n=383, 19.7%), failed induction of labor (n=106, 5.5%), or unsuccessful operative vaginal delivery (n=11, 0.6%).

With regard to the cumulative number of neighborhood SDoH, individuals exposed to only one (adjusted relative risk [RR] 1.14, 95% CI, 1.01–1.27) and two or more (adjusted RR 1.19, 95% CI, 1.06–1.34) adverse neighborhood SDoH measures compared with none had a higher risk of NTSV cesarean delivery (Table 2). When further evaluating specific neighborhood SDoH combinations, we found that only low walkability (adjusted RR 1.17, 95% CI, 1.06–1.28), low walkability and high ADI (adjusted RR 1.21, 95% CI, 1.03–1.41), and low walkability and low food access (adjusted RR 1.20, 95% CI, 1.05–1.37) were associated with an increased risk of cesarean delivery.

**Table 2.** Association of Intersections of Neighborhood Social Determinants of Health Measures With Cesarean Delivery Among Nulliparous, Term, Singleton, Vertex Pregnancies

	Cesarean Delivery (Row Percentage)			Unadjusted and Adjusted Analyses	
	Overall (N=8,010)	Yes (n=1,944)	No (n=6,066)	Unadjusted RR (95% CI)	Adjusted RR (95% CI)*
Primary analysis					
Combination of neighborhood SDoH measures					
None	1,871	419 (22.4)	1,452 (77.6)	1.00	1.00
Only 1	3,162	761 (24.1)	2,401 (75.9)	1.07 (0.97–1.19)	**1.14 (1.01–1.27)**
2 or more	2,977	764 (25.7)	2,213 (74.3)	**1.15 (1.03–1.27)**	**1.19 (1.06–1.34)**
Secondary analysis					
Combination of specific neighborhood SDoH measures					
None	1,871	419 (22.4)	1,452 (77.6)	1.00	1.00
Only high ADI	631	133 (21.1)	498 (78.9)	0.94 (0.79–1.12)	1.04 (0.85–1.27)
Only low walkability	2,394	599 (25.0)	1,795 (75.0)	1.12 (1.00–1.25)	**1.16 (1.04–1.31)**
Only low food access	137	29 (21.2)	108 (78.8)	0.95 (0.68–1.32)	0.94 (0.64–1.39)
High ADI+low walkability	1,158	326 (28.2)	832 (71.8)	**1.26 (1.11–1.42)**	**1.21 (1.03–1.41)**
High ADI+low food access	68	14 (20.6)	54 (79.4)	0.92 (0.57–1.48)	1.06 (0.64–1.73)
Low walkability+low food access	1,419	350 (24.7)	1,069 (75.3)	1.10 (0.97–1.25)	**1.20 (1.05–1.37)**
High ADI+low walkability+low food access	332	74 (22.3)	258 (77.7)	1.00 (0.80–1.24)	1.06 (0.84–1.35)
Individual neighborhood SDoH measures					
ADI					
Tertile 1 (least deprivation)	3,605	875 (24.3)	2,730 (75.7)	1.00	1.00
Tertile 2	2,216	522 (23.6)	1,694 (76.4)	0.97 (0.88–1.07)	0.98 (0.88–1.08)
Tertile 3 (most deprivation)	2,189	547 (25.0)	1,642 (75.0)	1.03 (0.94–1.13)	1.00 (0.89–1.12)
Low walkability					
No	2,707	595 (22.0)	2,112 (78.0)	1.00	1.00
Yes	5,303	1,349 (25.4)	3,954 (74.6)	**1.16 (1.06–1.26)**	**1.17 (1.06–1.28)**
Low food access					
No	6,054	1,477 (24.4)	4,577 (75.6)	1.00	1.00
Yes	1,956	467 (23.9)	1,489 (76.1)	0.98 (0.89–1.07)	1.04 (0.95–1.15)

RR, risk ratio; SDoH, social determinants of health; ADI, Area Deprivation Index.

Data are n (%) unless otherwise specified.

Bold indicates statistically significant (*P*<.05).

* Models adjusted for age, insurance status, body mass index, chronic hypertension, and pregestational diabetes.

In secondary analyses, the distribution of indications for cesarean delivery did not differ by different combinations of neighborhood SDoH measures (Appendix 3, https://links.lww.com/AOG/E775). Next, when neighborhood SDoH measures were assessed separately, low walkability (adjusted RR 1.17, 95% CI, 1.06–1.28), but not high ADI or low food access, was associated with a statistically higher risk of NTSV cesarean delivery (Table 2). Finally, there was no evidence of effect modification of the association between neighborhood SDoH and cesarean delivery by race and ethnicity, such that this association was similar across subgroups (interaction *P*=.69 in the multivariable regression model).

## DISCUSSION

In a prospective U.S. cohort, living in a neighborhood with low walkability, alone and in combination with socioeconomic disadvantage and food access, was associated with an increased risk of NTSV cesarean delivery. More broadly, living in a neighborhood with one or two or more adverse neighborhood SDoH measures compared with none was associated with an increased risk of NTSV cesarean delivery. Although the effect estimates may appear modest, given that neighborhood-level SDoH are experienced by a broader community, even a modest effect estimate can have a significant population-level effect. There was no effect modification by self-reported racial and ethnic subgroup for this association or differences in clinical indications for cesarean delivery among various combinations of neighborhood SDoH.

These results build on prior work that demonstrated the relative overlap of multiple neighborhood SDoH measures in early pregnancy with each other^12^ but did not examine how such overlap may be associated with pregnancy outcomes. In addition, these results that examine the potential influence of combinations of neighborhood SDoH measures build on demonstrated associations between each of the three neighborhood SDoH measures assessed individually and pregnancy outcomes, including the ADI and its associations with abnormal birth weight, stillbirth, postpartum readmission, and predicted risk of cardiovascular disease^19–21^; low food access with periconceptional diet and glycemic control^10,22^; and low walkability and glycemic control and gestational weight gain.^11,23^ With regard to the outcome of NTSV cesarean delivery, prior data have examined its association with individual SDoH measures assessed separately such as socioeconomic status, educational attainment, and employment status,^5^ and of note, none of the neighborhood SDoH measures assessed in the current study have been previously examined with NTSV cesarean delivery.

Racially and ethnically minoritized individuals were more likely to experience adverse neighborhood SDoH measures, which is consistent with prior studies.^24,25^ In the present analysis, the association between adverse neighborhood SDoH and cesarean delivery was similar across race and ethnicity, demonstrating how disparate exposure may underlie persistent disparities in maternal health and cesarean delivery.

Further research that evaluates how the intersection of multiple and concurrent neighborhood SDoH contributes to adverse pregnancy outcomes is needed.^26,27^ For example, additional neighborhood SDoH measures should be examined, including those thematic areas highlighted in Healthy People 2030 such as maternity care deserts and redlining. In addition, the overlap between individual and neighborhood SDoH concurrently and these SDoH intersections in relation to pregnancy outcomes should be investigated. Second, given the persistent rise and high rate of NTSV cesarean delivery in the United States, current recommendations emphasize the use of standardized guidance for labor management and clinical indications for labor cesarean delivery.^17,28^ The data in the present study were collected before these national efforts,^17^ and whether standardized guidance may have affected these findings requires further study. In the present analysis, clinical indications did not vary by neighborhood SDoH, and whether current recommendations are used differentially by clinicians and patients according to patient lived experience requires investigation. Third, efforts to address the disparate experiences of adverse neighborhood SDoH among racially and ethnically minoritized groups for the safe prevention of the first cesarean require further study. Finally, data from the present study could inform electronic health record dashboards that display standardized measures of SDoH and that could be used to improve delivery experience, particularly among minoritized groups.^29^

Strengths of this study include the use of a novel approach to understand the intersectional or overlapping relationship between three commonly measured neighborhood SDoH measures with a clinically important obstetric and patient-reported outcome, NTSV cesarean delivery. These results are likely generalizable given the use of data from a diverse prospective U.S. nulliparous pregnancy cohort.

There are study limitations to note. There are additional SDoH measures across other Healthy People 2030 SDoH domains such as education access and quality, health care access and quality, or social and community context that were not assessed in this study. We assessed the overlap between three neighborhood SDoH measures but did not integrate additional SDoH measures that could also influence an individual's risk of cesarean delivery. For example, further pregnancy data are needed to understand how measures of physical activity may intersect with walkability and food insecurity with low food access.^22^ We also could not account for differences in environmental exposure for individuals moving to different neighborhoods during their pregnancy because only first-trimester addresses were used to define neighborhood SDoH metrics in the original study.^30^ Given that excluded individuals were primarily missing neighborhood SDoH data to geocode and were more likely to experience individual SDoH factors, the present analysis likely underestimated the association between neighborhood SDoH and cesarean delivery (ie, nondifferential misclassification). This study was exploratory in nature; thus, no adjustments were made for multiple comparisons. Lastly, although these data are now a decade old, these results should remain generalizable because it is unlikely that the underlying associations between SDoH factors and obstetric outcomes have changed, and in fact, such differences may be more pronounced given the persistence of and increase in adverse neighborhood SDoH in many regions.^31^

In a prospective U.S. cohort, living in a neighborhood with low walkability, alone and in combination with socioeconomic disadvantage and food access, was associated with an increased risk of NTSV cesarean delivery. This association was similar for all racial and ethnic groups, which, given the differential exposure to adverse neighborhood SDoH, demonstrates how differences in neighborhood resources can translate to racial disparities in pregnancy outcomes. Further research that evaluates how the intersection of multiple SDoH measures contributes to adverse obstetric outcomes may be used to develop multilevel structural interventions to improve maternal health.

## References

[1] Osterman MJK. Changes in primary and repeat cesarean delivery: United States 2016-2021. NCHS Vital Statistics rapid release reports 2022; report number 21. Accessed December 1, 2025. https://stacks.cdc.gov/view/cdc/117432

[2] Sharma G Grandhi GR Acquah I Mszar R Mahajan S Khan SU . Social determinants of suboptimal cardiovascular health among pregnant women in the United States. J Am Heart Assoc 2022;11:e022837. doi: 10.1161/JAHA.121.022837 35014862 PMC9238529

[3] Okwandu IC Anderson M Postlethwaite D Shirazi A Torrente S. Racial and ethnic disparities in cesarean delivery and indications among nulliparous, term, singleton, vertex women. J Racial Ethnic Health Disparities 2022;9:1161–71. doi: 10.1007/s40615-021-01057-w PMC9249704 34254270

[4] Gibson K Bailit JL. Cesarean delivery as a marker for obstetric quality. Clin Obstet Gynecol 2015;58:211–6. doi: 10.1097/grf.0000000000000107 25860325

[5] Amjad S MacDonald I Chambers T Osornio‐Vargas A Chandra S Voaklander D Social determinants of health and adverse maternal and birth outcomes in adolescent pregnancies: a systematic review and meta-analysis. Paediatr Perinat Epidemiol 2019;33:88–99. doi: 10.1111/ppe.12529 30516287

[6] Healthy people 2030. Social Determinants of Health. Accessed January 4, 2024. https://health.gov/healthypeople/priority-areas/social-determinants-health

[7] Kershaw KN Magnani JW Diez Roux AV Camacho-Rivera M Jackson EA Johnson AE Neighborhoods and cardiovascular health: a scientific statement from the American Heart Association. Circ Cardiovasc Qual Outcomes 2024;17:e000124. doi: 10.1161/hcq.0000000000000124 38073532

[8] Venkatesh KK Germann K Joseph J Kiefer M Buschur E Thung S Association between social vulnerability and achieving glycemic control among pregnant individuals with pregestational diabetes. Obstet Gynecol 2022;139:1051–60. doi: 10.1097/aog.0000000000004727 35675602 PMC10953616

[9] Meiman J Grobman WA Haas DM Yee LM Wu J McNeil B . Association of neighborhood socioeconomic disadvantage and postpartum readmission. Obstet Gynecol 2023;141:967–70. doi: 10.1097/AOG.0000000000005151 37026732 PMC10147577

[10] Venkatesh KK Joseph JJ Clark A Gabbe SG Landon MB Thung SF Association of community-level food insecurity and glycemic control among pregnant individuals with pregestational diabetes. Prim Care Diabetes 2023;17:73–8. doi: 10.1016/j.pcd.2022.11.002 36379871 PMC10286113

[11] Field C Lynch CD Fareed N Joseph JJ Wu J Thung SF Association of community walkability and glycemic control among pregnant individuals with pregestational diabetes mellitus. Am J Obstet Gynecol MFM 2023;5:100898. doi: 10.1016/j.ajogmf.2023.100898 36787839 PMC12805509

[12] Hamilton JL Grobman WA Wu J Yee LM Haas D Mcneil B Intersectionality of individual and neighborhood-level adverse social determinants of health in early pregnancy. Pregnancy 2025;1:e70002. doi: 10.1002/pmf2.70002 PMC13344772 42597062

[13] Haas DM Parker CB Wing DA Parry S Grobman WA Mercer BM A description of the methods of the Nulliparous Pregnancy Outcomes Study: Monitoring Mothers-to-Be (nuMoM2b). Am J Obstet Gynecol 2015;212:539.e1–24. doi: 10.1016/j.ajog.2015.01.019 PMC4387083 25648779

[14] Rhone A Ver Ploeg M Dicken C Williams R Breneman V. Low-income and low-supermarket-access census tracts, 2010-2015. Econ Inf Bull 2017;165:1–15. Accessed February 1, 2026. https://ers.usda.gov/publications/82100

[15] U.S. Department of Agriculture. Introduction to the Food Research Atlas. Accessed February 1, 2026. sportal.ers.usda.gov/portal/apps/experiencebuilder/experience/?id=a53ebd7396

[16] U.S. Environmental Protection Agency. National Walkability Index methodology and user guide. Accessed February 1, 2026. https://www.epa.gov/sites/default/files/2021-06/documents/national_walkability_index_methodology_and_user_guide_june2021.pdf

[17] Safe prevention of the primary cesarean delivery. Obstetric Care Consensus No. 1. American College of Obstetricians and Gynecologists. Obstet Gynecol 2014;123:693–711. doi: 10.1097/01.AOG.0000444441.04111.1d 24553167

[18] Fleischer N Roux AVD. Using directed acyclic graphs to guide analyses of neighbourhood health effects: an introduction. J Epidemiol Community Health 2008;62:842–6. doi: 10.1136/jech.2007.067371 18701738

[19] Venkatesh KK Yee LM Johnson J Wu J McNeil B Mercer B . Neighborhood socioeconomic disadvantage and abnormal birth weight. Obstet Gynecol 2023;142:1199–207. doi: 10.1097/AOG.0000000000005384 37769319 PMC10972636

[20] Hajmurad S Grobman WA Haas DM Yee LM Wu J McNeil B . Fetal death and neighborhood socioeconomic disadvantage. Am J Obstet Gynecol 2024;230:e86–91. doi: 10.1016/j.ajog.2023.12.016 38360448

[21] Venkatesh KK Khan SS Catov J Wu J McNeil R Greenland P . Socioeconomic disadvantage in pregnancy and postpartum risk of cardiovascular disease. Am J Obstet Gynecol 2025;232:226.e1–14. doi: 10.1016/j.ajog.2024.05.007 38759711

[22] Venkatesh K Walker DM Yee LM Wu J Garner J McNeil B Association of living in a food desert and poor periconceptional diet quality in a cohort of nulliparous pregnant individuals. J Nutr 2023;153:2432–41. doi: 10.1016/j.tjnut.2023.06.032 37364682 PMC10447609

[23] Grobman WA Crenshaw EG Marsh DJ McNeil RB Pemberton VL Haas DM Associations of the neighborhood built environment with gestational weight gain. Am J Perinatol 2023;40:638–45. doi: 10.1055/s-0041-1730363 34082443 PMC8697035

[24] Salow AD Pool LR Grobman WA Kershaw KN. Associations of neighborhood-level racial residential segregation with adverse pregnancy outcomes. Am J Obstet Gynecol 2018;218:351.e1–7. doi: 10.1016/j.ajog.2018.01.022 29421603

[25] van Daalen KR Kaiser J Kebede S Cipriano G Maimouni H Olumese E Racial discrimination and adverse pregnancy outcomes: a systematic review and meta-analysis. BMJ Glob Health 2022;7:e009227. doi: 10.1136/bmjgh-2022-009227 PMC9344988 35918071

[26] Figueroa JF Frakt AB Jha AK. Addressing social determinants of health: time for a polysocial risk score. JAMA 2020;323:1553–4. doi: 10.1001/jama.2020.2436 32242887

[27] Javed Z Valero-Elizondo J Dudum R Khan SU Dubey P Hyder A Development and validation of a polysocial risk score for atherosclerotic cardiovascular disease. Am J Prev Cardiol 2021;8:100251. doi: 10.1016/j.ajpc.2021.100251 34553187 PMC8441152

[28] Spong CY Berghella V Wenstrom KD Mercer BM Saade GR. Preventing the first cesarean delivery: summary of a joint *Eunice Kennedy Shriver* National Institute of Child Health and Human Development, Society for Maternal-Fetal Medicine, and American College of Obstetricians and Gynecologists workshop. Obstet Gynecol 2012;120:1181–93. doi: 10.1097/aog.0b013e3182704880 23090537 PMC3548444

[29] Metwali NY Ahmed RA Hussain TJ Irfan H Makarfi SM Metwali MY Evidence-based strategies to minimize unnecessary primary cesarean sections: a comprehensive review. Cureus 2024;16:e74729. doi: 10.7759/cureus.74729 39735004 PMC11682606

[30] Bell ML Belanger K. Review of research on residential mobility during pregnancy: consequences for assessment of prenatal environmental exposures. J Expo Sci Environ Epidemiol 2012;22:429–38. doi: 10.1038/jes.2012.42 22617723 PMC3543155

[31] Paremoer L Nandi S Serag H Baum F. Covid-19 pandemic and the social determinants of health. BMJ 2021;372:n129. doi: 10.1136/bmj.n129 33509801 PMC7842257

